# A 12-month follow-up of an influenza vaccination campaign based on voluntary adherence: report on upper-respiratory symptoms among volunteers and non-volunteers

**DOI:** 10.1590/S1516-31802001000400006

**Published:** 2001-07-07

**Authors:** Páris Ali Ramadan, Francisco Barreto de Araújo, Mario Ferreira

**Keywords:** Flu, Influenza, Vaccine, Gripe, Influenza, Vacina

## Abstract

**CONTEXT:**

Routine immunization of groups at high risk for influenza has been progressively implemented as a matter of Brazilian public health policy. Although the benefits of the vaccination for healthy young adults are still controversial, it has been offered yearly to hundreds of thousands of Brazilian workers, generally as part of wellness initiatives in the workplace.

**OBJECTIVE:**

To study the characteristics of subjects that accepted or refused to be vaccinated against influenza and to report on respiratory symptoms in both groups, one year after the campaign date.

**DESIGN:**

A prospective observational study.

**SETTING:**

Workersat a subsidiary of an international bank in São Paulo, Brazil.

**PARTICIPANTS:**

124 persons that did not accept and 145 that voluntarily accepted the vaccine completed 12 months of follow-up.

**MAIN MEASUREMENTS:**

Data concerning gender, age, tobacco use, and any history of chronic respiratory illness such as asthma, bronchitis, rhinitis, and repetitive upper-respiratory infections, were recorded at the time of vaccination. After that, workers were asked monthly by questionnaire or telephone about respiratory symptoms, days of work lost and medical consultations.

**RESULTS:**

The results showed statistically significant differences regarding age (P = 0.004) with the vaccinated group (V) being younger than the non-vaccinated (NV) one, and with reference to previous repetitive upper-respiratory infections being higher among the V group (P < 0.0001). During the follow-up, the V group reported more occurrences of upper respiratory symptoms (P < 0.0001), due to both non-influenza (P < 0.0001) and influenza-like illness (P = 0.045). Differences were also found between V and NV groups concerning days off work and number of medical consultations due to upper-respiratory symptoms and non-influenza illness. Gender and history of repetitive upper-respiratory infections were the best predictors of influenza-like illness-related events.

**CONCLUSIONS:**

The making of previous reference to repetitive upper-respiratory infections was a major difference between those who accepted or rejected the vaccine. The vaccination itself was not sufficient to reduce the number of occurrences of respiratory symptoms and related absenteeism to levels similar to those found among non-vaccinated people.

## RESUMO

**CONTEXTO:**

A imunização de rotina de grupos de alto risco para Influenza vem sendo progressivamente implantada como parte de políticas públicas de saúde no Brasil. Embora os benefícios da vacinação de indivíduos jovens saudáveis ainda sejam controvertidos, a vacina tem sido oferecida anualmente a centenas de milhares de trabalhadores brasileiros, em geral, como parte de programas de qualidade de vida em locais de trabalho.

**OBJETIVO:**

Estudar as características dos indivíduos que aceitaram ou recusaram a vacina contra Influenza, e a referência a apresentação de sintomas respiratórios em ambos os grupos por um ano após a data da campanha.

**TIPO DE ESTUDO:**

Estudo observacional prospectivo.

**LOCAL:**

Trabalhadores de uma filial de banco internacional em São Paulo, Brasil.

**AMOSTRA:**

124 pessoas que não aceitaram a vacina e 145 que a aceitaram e completaram os 12 meses de acompanhamento

**VARIÁVEIS ESTUDADAS:**

Dados relativos a sexo, idade, consumo de tabaco, história de doenças respiratórias crônicas, como asma, bronquite, rinite, e infecções repetidas de vias aéreas superiores, foram coletados no momento da vacinação. Após, os trabalhadores foram contatados mensalmente por questionário ou telefone a fim de se obter informações sobre sintomas, perda de dias de trabalho e consultas médicas por problemas respiratórios.

**RESULTADOS:**

Os resultados mostraram diferenças significativas em relação a idade, sendo o grupo vacinado (V) mais jovem (P = 0.004), e história de infecções respiratórias repetitivas, predominantes no grupo V (P < 0.0001). Durante o acompanhamento, o grupo V referiu mais sintomas de vias aéreas superiores (P < 0.0001), assim como mais ocorrências de quadros não compatíveis (P < 0.0001) ou compatíveis com gripe (P = 0.045). O grupo V ausentou-se mais ao trabalho e procurou mais consultas médicas por sintomas de vias aéreas superiores e quadros não compatíveis com gripe. Sexo e história pregressa de infecções de vias aéreas superiores foram os melhores "preditores" dos eventos associados aos quadros compatíveis com gripe.

**CONCLUSÃO:**

A referência prévia a episódios repetitivos de infecções de vias aéreas superiores foi uma diferença marcante entre quem aceitou ou rejeitou a vacina. A vacinação isoladamente não foi suficiente para reduzir o número de ocorrências de sintomas respiratórios e o absenteísmo a eles relacionados a níveis semelhantes aos encontrados entre os não vacinados.

## INTRODUCTION

The effectiveness of annual vaccination against influenza is relatively well established on the basis of good evidence in reducing morbidity and mortality due to the infection itself and its complications.^1^ Such vaccination is targeted on health care professionals and patients at high risk, such as the elderly (over 65 years), people who have chronic respiratory, cardiac or renal illnesses, diabetes, or those that are immunocompromised. Routine immunization of these groups has been progressively implemented as a matter of Brazilian public health policy.^2^

Although the benefits of the vaccination for healthy young adults are still controversial,^3^ it has been offered yearly to hundreds of thousands of Brazilian workers, generally as an initiative in wellness programs in the workplace. The estimated efficacy of influenza vaccine is about 70 to 86%,^4^ and some published data have supported the vaccination of the workforce because of possible reduction in incidence, absenteeism, and cost associated with influenza.^5,6^ However, a recently published randomized controlled trial, comparing the results from influenza vaccine to placebo during two consecutive years, has shown that the vaccination may not be effective every year and that no economic benefit may be achieved even when the virus samples included in the vaccine match the circulating virus.^7^

The vaccine is only able to provide avoidance of influenza and its complications. Nonetheless, the general expected effects of vaccination can easily be overestimated by patients, especially due to lack of information. As a matter of fact, the time or productivity losses at work are a general result of upper-respiratory symptoms caused by several agents or allergies. Moreover, the effectiveness of protection against influenza is directly dependent on the virus-matching of the produced samples, the intensity of virus circulation and the characteristics inherent to individuals or groups.

The proper evaluation of each factor can be decisive in the vaccination strategy to be stimulated: an open campaign with voluntary adherence vs. targeting on people at increased risk. To this end, through an observational study, we recorded some characteristics of subjects that accepted being vaccinated against influenza (group V) and other non-vaccinated ones (group NV), and reported on respiratory symptoms in both groups during one year of follow-up.

## METHODS

In April 1998, vaccine against influenza was offered as part of a wellness program for workers at a subsidiary of an international bank in São Paulo, Brazil. The vaccine samples were those available in the market from the major worldwide manufacturers, and were in accordance with WHO recommendations. Staff at the bank's clinic were in charge of all precautions related to sample handling, from reception to administration.

About 500 out of 2054 workers accepted the vaccine. Then, 237 V and 399 NV persons working under similar conditions were randomly contacted for participation in a follow-up scheduled over the next 12 months (from May 1998 to April 1999). The followup inquired about the appearance of upper- respiratory symptoms (URS) divided into two subsets: influenza-like illness (ILI) or non-influenza illness (NII). ILI was defined as the simultaneous presence of at least two upper-respiratory symptoms (nasal obstruction, coryza, sore throat, cough) with at least one systemic complaint (fever, myalgia).^5^

Data concerning gender, age, tobacco use, and history of chronic respiratory illness (CRI) such as asthma, bronchitis, rhinitis, and repetitive upper-respiratory infections (RUI), defined by reporting two or more respiratory infections per year, were recorded at the time of vaccination. After that, workers were assessed monthly by questionnaire or telephone inquiring about symptoms, days of work lost and medical consultations. Of the initially contacted workers, 124 NV (31.1%) and 145 V (61.2%) completed the twelve monthly follow-ups. The health personnel were also in charge of the survey and contacts.

All data collected was utilized to form a database that was statistically analyzed with SPSS 8.0 software. Descriptive statistics included the chi-squared test with Pearson's test and Student's t-test for comparison between means. Multivariate linear regression was done to identify predictors for respiratory symptoms and their related outcomes. The level of significance of 0.05 was used to define differences between groups and means and the major predictors in statistical models.

## RESULTS

The comparisons of the general data obtained at the time of vaccination are depicted in Table 1. The results showed statistically significant differences in relation to age (P = 0.004, with the V group younger than the NV), and the reference to previous repetitive upper-respiratory infections (RUI), which was higher among the V group (P < 0.0001).

**Table 1:** Characteristics of studied groups, vaccinated (V) and non-vaccinated (NV), regarding demographic data, report of previous respiratory illnesses and tobacco use, and comparison between groups

Population	Vaccinated (V) N=145	Non-vaccinated (NV) N=124	P
Gender (Female)	81	57	NS^3^
Age	30.21 (6.68)^1^	32.61 (6.72)	0.004^2^
CRI (Yes)	37	26	NS^3^
RUI (Yes)	100	57	<0.0001^3^
Tobacco use (Yes)	19	24	NS^3^
Packet-year of cigarettes	1.23 (4.16)	1.45 (3.91)	NS^2^

CRI = chronic respiratory illness; RUI = repetitive upper-respiratory infections

1 Mean and Standard Deviation

2 Statistics obtained from Student's t-test

3 Statistics obtained from chi-squared test with Pearson's exact test.

Table 2 presents comparisons of URS, ILI and NII occurrences and their consequences (days off work and number of medical consultations) between V and NV groups. The results indicated that the V group reported more URS (P < 0.0001), NII (P < 0.0001) and ILI (P = 0.045) occurrences than did the NV group, during the year of follow-up. Differences were also found between groups in relation to days off work (P = 0.002) and number of medical consultations (P = 0.02) due to NII, with higher numbers in the V group. URS caused more work absence among V than NV individuals (P = 0.037).

**Table 2:** Outcomes related to upper-respiratory symptoms (URS) and subsets, influenza-like illness (ILI) and non-influenza illness (NII), and comparison between the vaccinated (V) and non-vaccinated (NV) groups #

	Vaccinated (V) N=145	Non-vaccinated (NV) N=124	P*
**URS-related events per 12 months**			
• No. of occurrences	4.21 (2.51)	2.64 (2.00)	<0.0001
• No. of days off work	0.68 (1.24)	0.36 (1.26)	0.037
• No. of medical consultations	1.20 (1.56)	0.85 (1.42)	NS
**ILI-related events per 12 months**			
• No. of occurrences	1.59 (1.47)	1.24 (1.38)	0.045
• No. of days off work	0.57 (1.14)	0.35 (1.26)	NS
• No. of medical consultations	0.83 (1.18)	0.69 (1.32)	NS
**NII-related events per 12 months**			
• No. of occurrences	2.62 (2.31)	1.40 (1.39)	<0.0001
• No. of days off work	0.12 (0.42)	0.008 (0.089)	0.002
• No. of medical consultations	0.37 (0.90)	0.16 (0.50)	0.02

# All data are presented as Mean and Standard Deviation

* All statistics were obtained through Student's t test; NS = Not Significant.

Multiple linear regression models made for estimating the impact of predictors on ILI and NII-related outcomes are described in Table 3. A report of RUI was predictive of ILI-related days off work (P = 0.012) and medical consultations (P = 0.05), and NII occurrences (P = 0.04). Women were likely to present more ILI-related events.

**Table 3:** Results of multiple linear regression models applied to identify predictors of outcomes related to influenza-like illness (ILI) and non-influenza illness (NII), occurring during the 12-month follow-up.

Outcomes	Predictors							Model relevance
	Gender (M/F)	Age	RUI (N/Y)	CRI (N/Y)	P-Y Cig.	Vaccine (N/Y)	Constant	
**ILI-related events/12 months**								
• N of occurrences	b=0.435 P=0.013	NS	NS	NS	NS	NS	b=0.97 P=0.037	r^2^=0.077 P=0.002
• N of days off work	b=0.375 P=0.011	NS	b=0.39 P=0.012	NS	NS	NS	NS	r^2^=0.068 P=0.005
• N of medical consultations	b=0.392 P=0.01	NS	b=0.318 P=0.05	NS	NS	NS	NS	r^2^=0.07 P=0.004
**NII-related events/12 months**								
• No. of occurrences	NS	NS	b=0.52 P=0.04	NS	NS	b=1.07 P<0.0001	b=1.49 P=0.02	r^2^=0.12 P<0.0001
• No. of days off work	NS	NS	NS	NS	NS	b=0.09 P=0.02	NS	r^2^=0.04 (NS)
• No. of medical consultations	NS	NS	NS	NS	NS	b=0.18 P=0.055	b=0.54 P=0.031	r^2^=0.033 (NS)

CRI = chronic respiratory illness; RUI = repetitive upper-respiratory infections; P-Y cig. = packet-year of cigarettes; M = male; F = female; N = No; Y = Yes; NS = Not Significant.

## DISCUSSION

Vaccine is considered the best intervention for influenza prevention. However, while the benefits in reducing health complications and deaths among elderly people and those who have some chronic disease are well-established, its use among healthy young people is still controversial.^2^

The US Preventive Services Task Force reinforces the indication for groups at high risk as part of public health policy.^1^ On the contrary, the Centers for Disease Control and Prevention,^3^ Atlanta, recommend that the health care providers should stimulate the vaccination of their clients of any age and health status who worry about influenza infection. Since most experts agree about the efficacy of the vaccine for some population groups, the discussion should perhaps focus on vaccination strategy rather than on the efficacy itself.

As a matter of fact, the reason for offering the influenza vaccine to healthy workers has more likely been related to economic issues than health ones, since according to some authors the reduction of influenza-related absenteeism from work and the improvement of productivity would turn the initiative cost-effective.^6^ However, this was not confirmed in a recently published controlled trial that concluded that the benefits of vaccination can vary from year to year and it is possible that no money will be saved.^7^

The level of satisfaction with the results of vaccination depends on the degree of information about its benefits, complications and relapses. Since the infections to be prevented are only the ones caused by types of influenza viruses, a lack of clarification of the prevention limitations could seriously compromise the impact of the vaccination strategy, leading to an undesirable skepticism about outcomes and low adherence. This would probably be more hazardous in relation to the groups in which the vaccine is expected to be more effective.

This was an observational study designed to identify the characteristics of the people who voluntarily accepted the influenza vaccine and those who did not, during a campaign of vaccination, and to verify the differences in reporting respiratory symptoms and related outcomes during 12 months of follow-up. Comparing the basic characteristics of V to NV, a self-selection bias could be observed, as illustrated in Table 1, since the adherence to the vaccine was higher among the younger and those who reported previous history of RUI.

As a likely consequence of this selection bias, the statistics also showed that the number of occurrences of days off work and medical consultations related to URS, ILI or NII (Table 2) were higher in the V group.

According to these results the people who volunteered for the vaccination seemed to have greater prior susceptibility to respiratory symptoms, and this fact was confirmed during the follow-up when the differences remained despite the vaccination.

In the same way, multiple regression models (Table 3) revealed that female gender and positive previous history of RUI were the most important predictors of ILI-related outcomes in the evaluated population, suggesting that individual susceptibility to agents that cause upper-respiratory symptoms could influence the outcomes more than the influenza vaccination itself. However, the lack of statistical differences between the V and NV groups, in relation to the number of days off work and medical consultations related to ILI episodes, could represent lower morbidity promoted by the influenza vaccine.

In brief, this study has shown that the vaccine alone was not sufficient for reducing URS-related events and the number of ILI occurrences to levels similar to those presented by non-vaccinated persons, probably due to individual differences influencing the adherence to the vaccination campaign. This finding raises the question of the worth of stimulating voluntary adherence to influenza vaccination among healthy young people, without first taking a careful history of upper-respiratory symptoms and giving proper information about benefits, limitations and the most relevant recommendation criteria for the vaccine.

## CONCLUSION

The making of previous reference to repetitive upper-respiratory infections was a major difference between those who accepted or rejected the vaccine. The vaccination itself was not sufficient for reducing the number of occurrences of respiratory symptoms and related absenteeism to levels similar to those found among non-vaccinated people, and this fact was probably related to the differences previously observed between volunteers and non-volunteers.
